# Supplementary material for: Structural and mutational analysis of MazE6-operator DNA complex provide insights into autoregulation of toxin-antitoxin systems
Source: Commun Biol. 2022 Sep 15;5:963. doi: 10.1038/s42003-022-03933-5 (PMC9477884; doi:10.1038/s42003-022-03933-5)
Supplement: Supplementary file 2 — Supplementary Information [file 42003_2022_3933_MOESM2_ESM.pdf]

Structural and mutational analysis of MazE6-operator DNA complex provide insights into autoregulation of toxin-antitoxin systems

Khushboo Kumari and Siddhartha P Sarma*

Molecular Biophysics Unit, Indian Institute of Science, Bangalore, Karnataka, India

E-mail: sidd@iisc.ac.in

Phone: +91 80 2293 3454. Fax: +91 80 23600535

Running header

MazE6-operator DNA complex

*To whom correspondence should be addressed

Supplemetary Information

Table S1: DNA sequnces used in the study

Figure S1: Solution properties of MazE6

Figure S3: Sequence specific assignment of MazE6

Figure S4: Intrinsically disordered region of MazE6

Figure S5: Solution properties of nMazE6

Figure S6: Comparision of nMazE6 with MazE6

Figure S7: Interaction of nMazE6 with operator DNA:

Figure S8: Chemical Shift Perturbation (CSP) upon cognate operator DNA binding:

Figure S9: Chemical Shift Perturbation (CSP) upon non-cognate and mutated operator DNA binding:

Figure S10: Two-dimensional lineshape analysis of nMazE6 upon titration with operator DNA

Tables

Table S1: DNA sequences used in the study

**Name**	**DNA sequence (fwd)**	**dsDNA Mol. wt.(kDa)**
mazEF6 operator DNA	5′-CCGGTTATACTATCTGTA-3′	11.15
mazEF6 mutated-operator DNA	5′-CCGGTTAGCATATCTGTA-3′	11.15
Scrambled DNA	5′-TTTTCAACGGGTCCTTAA-3′	11.15
mazEF9 operator DNA	5′-TGGTAGCATCTTAGGTTG-3′	11.15

Figures

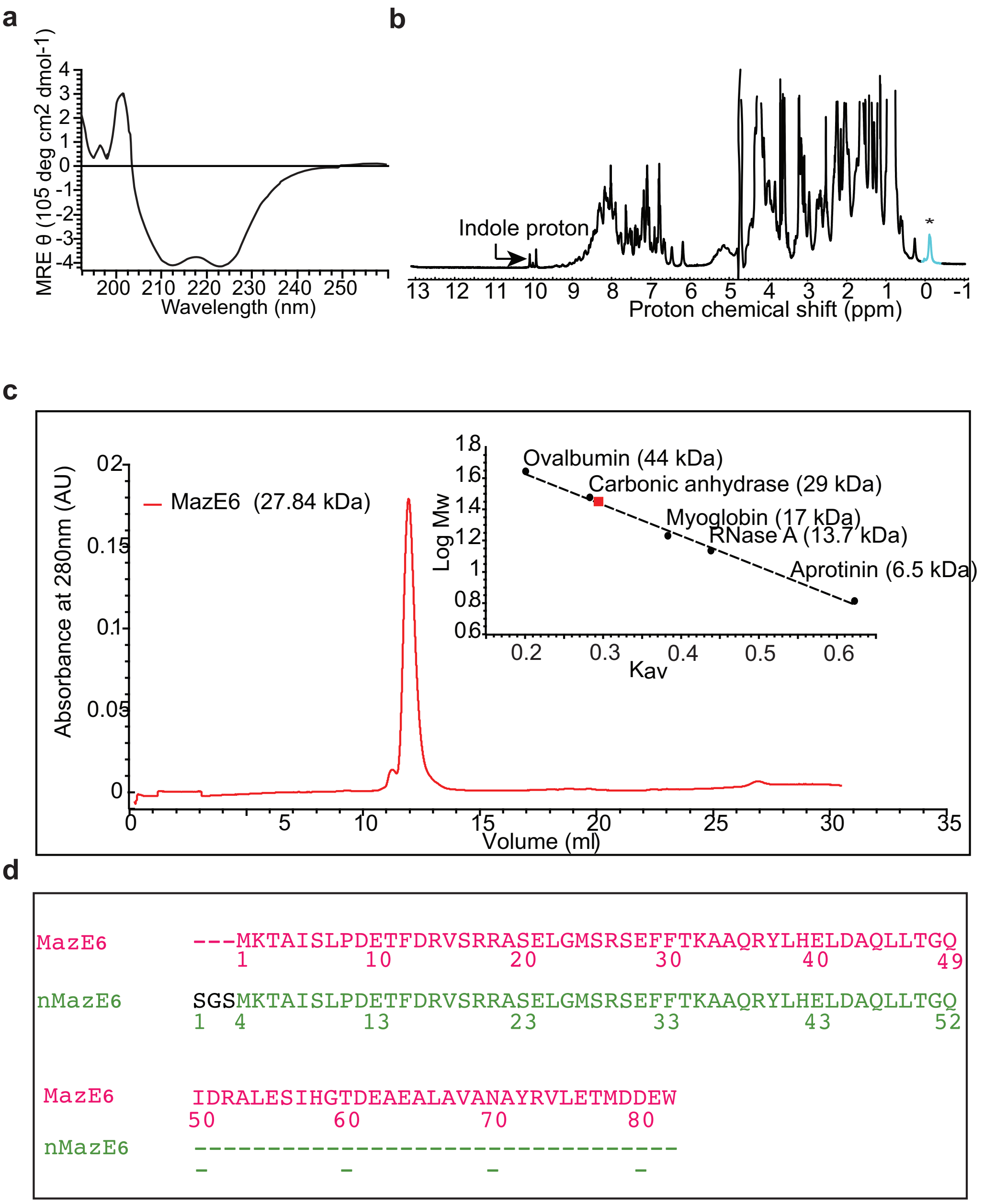

Figure S1

Figure S1: Solution properties of MazE6 (a) CD spectrum of MazE6 in phosphate buffer at pH 7 and 25°C. **(b)** 1D proton NMR spectrum of ^{15}N- MazE6. * indicates resonance lines that are upfield shifted (≤ 0 ppm) in MazE6. **(c)** Analytical size-exclusion chromatogram on S-75 analytical column. Inset shows the calibration curve for the column with the elution volumes of globular proteins of known molecular weight. The interpolated molecular weight of MazE6 is shown on the curve. **(d)** Sequence alignment of MazE6 and nMazE6. Initial three residues in nMazE6 are part of the expression tag.

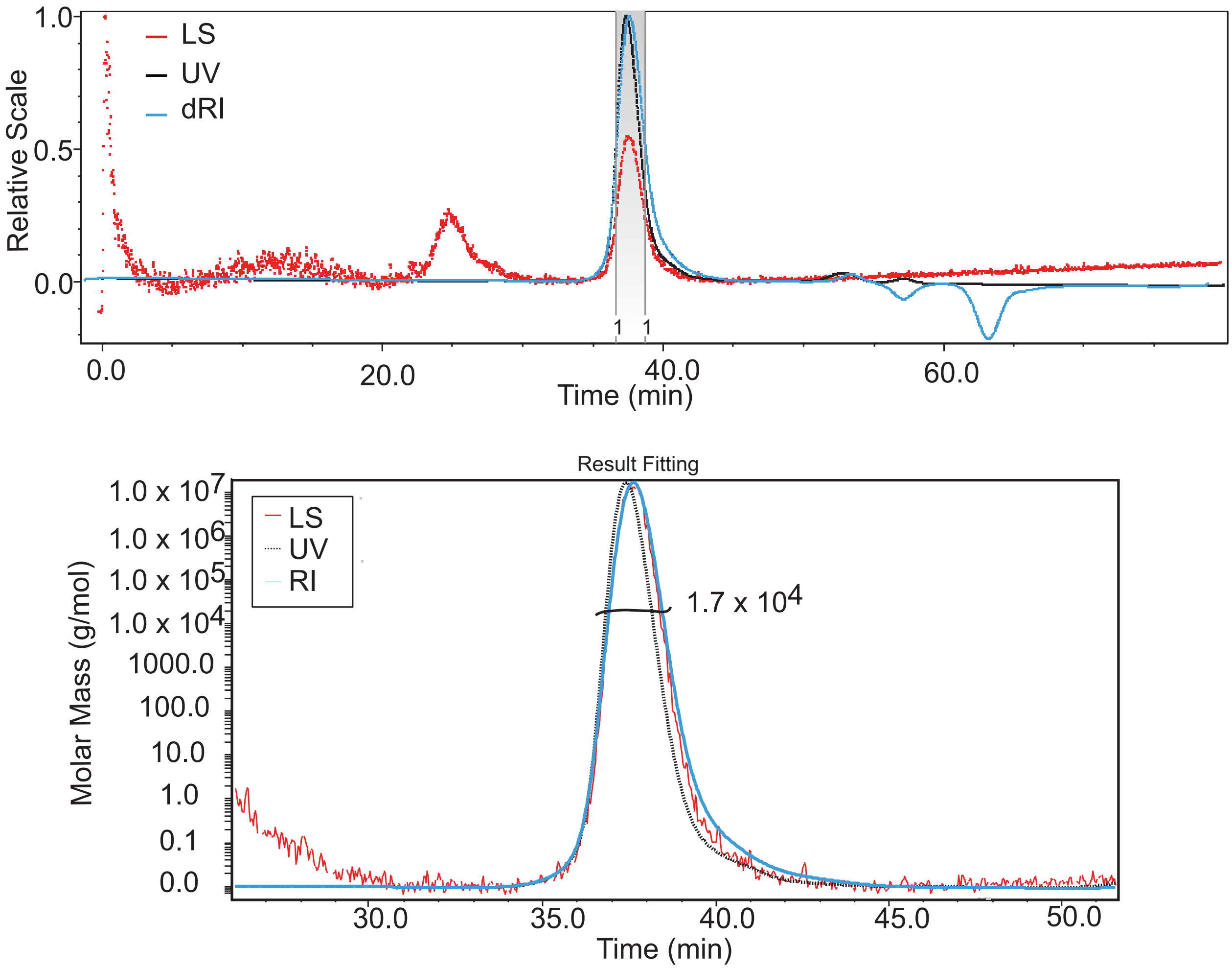

Figure S2

Figure S2: Oligomeric state of MazE6 Size Exclusion Chromatography- Multi Angle Light Scattering(SEC-MALS) profile of MazE6 showing the protein as dimer in solution.

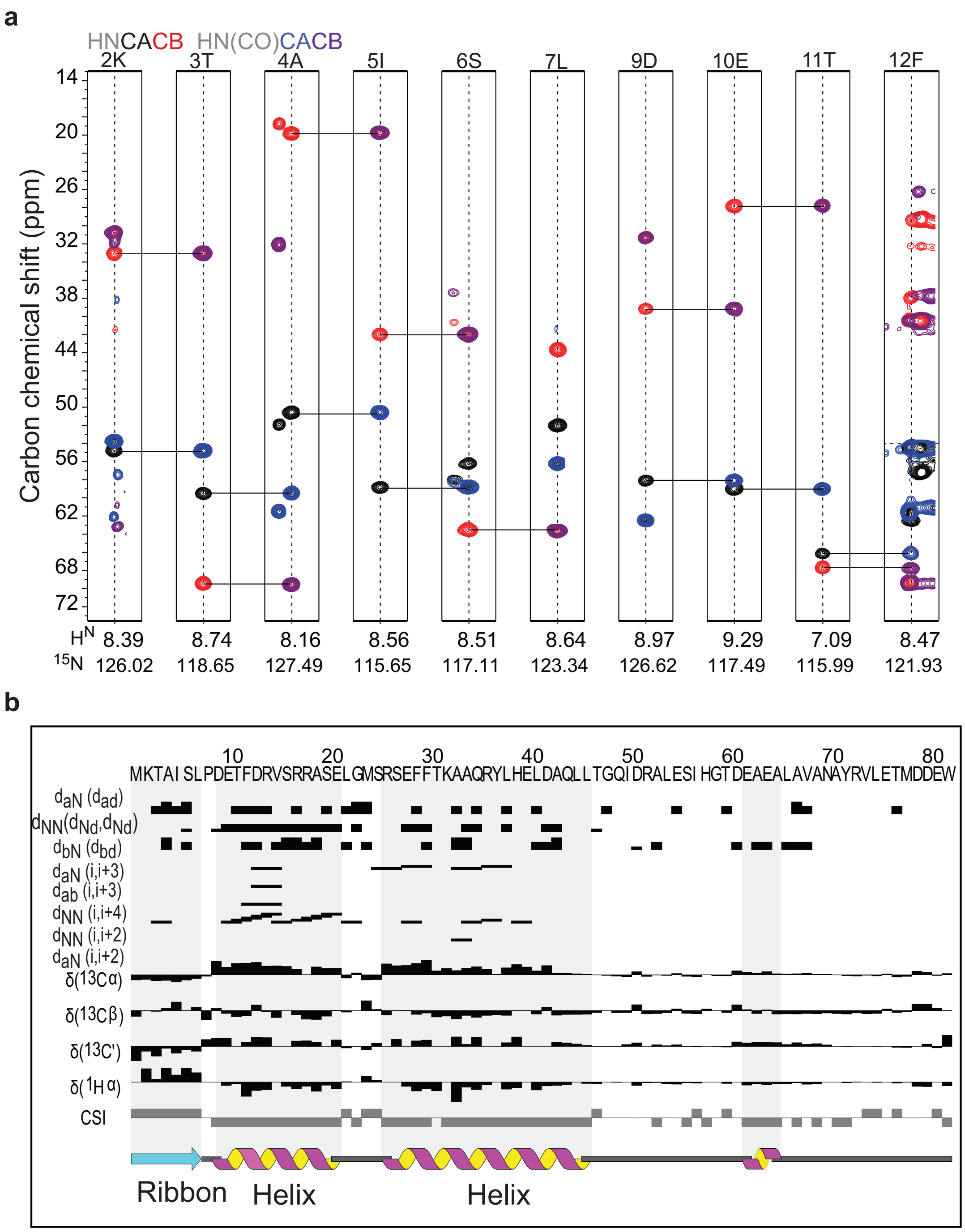

Figure S3

Figure S3: Sequence specific assignment of MazE6 (a) Overlay of strip-plots of HNCACB and HN(CO)CACB spectra of MazE6 showing sequential connectivity for residues 2 to 12. Data was acquired on $^{13}CH_3$- ILV, ^{2}H, ^{13}C, ^{15}N labeled samples of MazE6. **(b)** Secondary structure assignment for MazE6 based on secondary chemical shift values of ^{13}C, $^{13}C^{\alpha}$, $^{13}C^{\beta}$ and sequential-, short- and medium-range NOEs observed in MazE6.

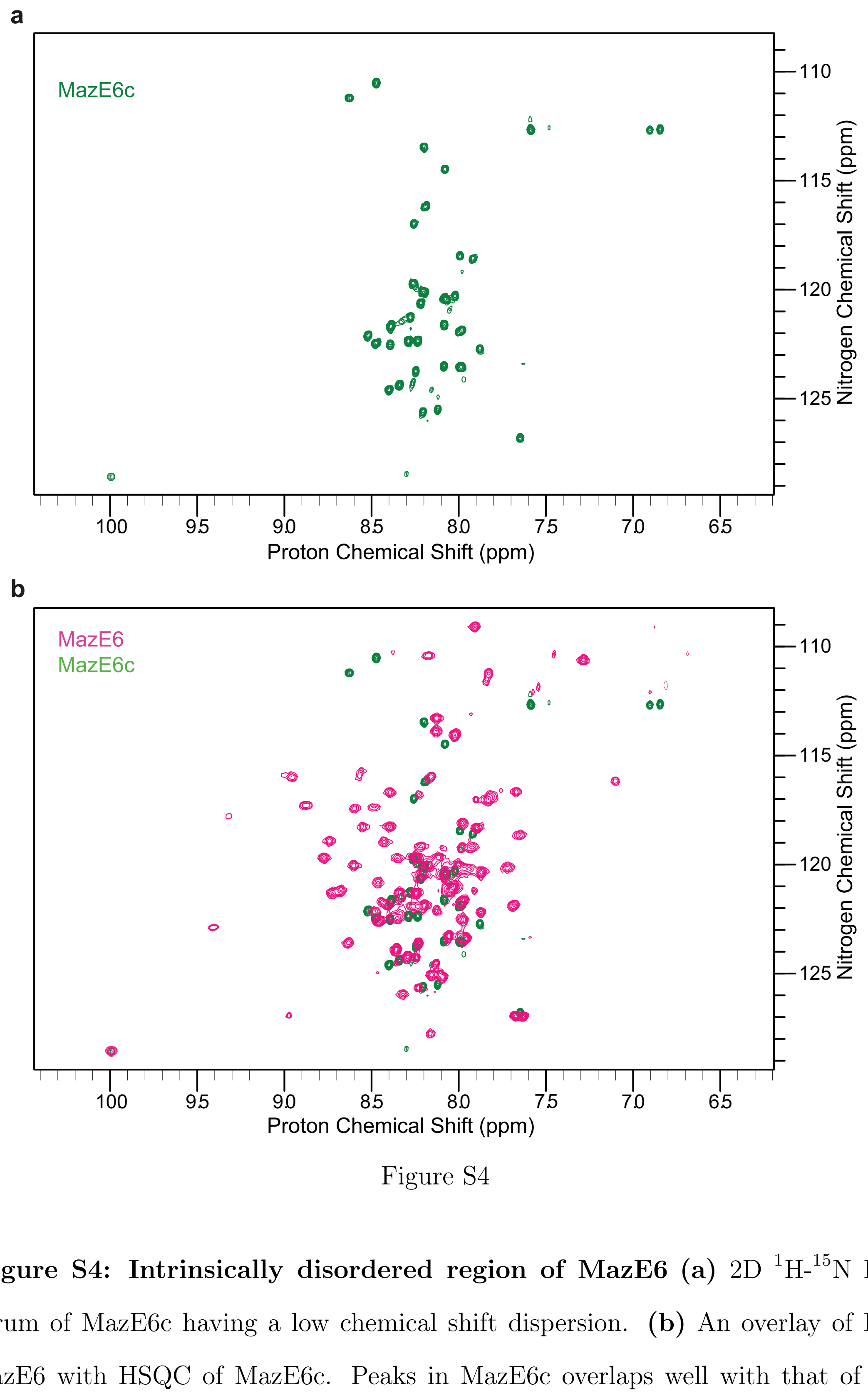

Figure S4

Figure S4: Intrinsically disordered region of MazE6 (a) 2D ^{1}H-^{15}N HSQC spectrum of MazE6c having a low chemical shift dispersion. **(b)** An overlay of HSQC of MazE6 with HSQC of MazE6c. Peaks in MazE6c overlaps well with that of peaks in disordered region of MazE6. Small shifts in the peak position are seen due to the difference in buffer conditions. MazE6 spectrum is acquired in phosphate buffer at pH 7 and MazE6c in acetate buffer at pH 5.5.

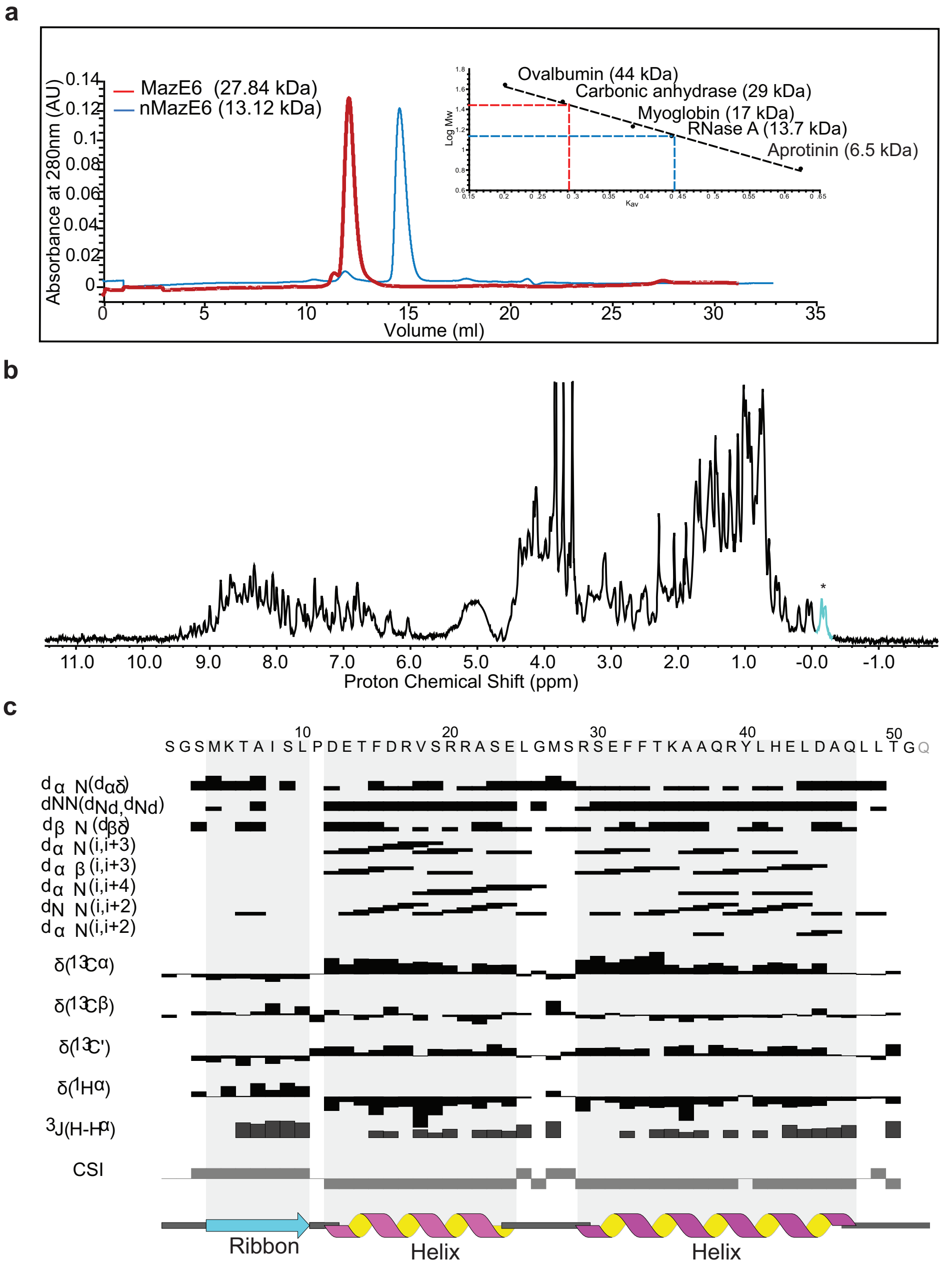

Figure S5

Figure S5: Solution properties of nMazE6 (a) Size exclusion chromatographs for MazE6 and nMazE6 on a Superdex75 analytical column. Inset shows the calibration curve for the column with the elution volumes of globular proteins of known molecular weight. The interpolated molecular weights of MazE6 and nMazE6 are shown on the curve. **(b)** 1D proton NMR spectrum of nMazE6 showing the presence of upfield shifted lines below 0 ppm (*). **(c)** Secondary structure assignment for nMazE6 based on secondary chemical shift values of $^{13}C'$, $^{13}C^{\alpha}$, $^{13}C^{\beta}$, CSI chart and sequential-, short- and medium-range NOEs

observed in nMazE6.

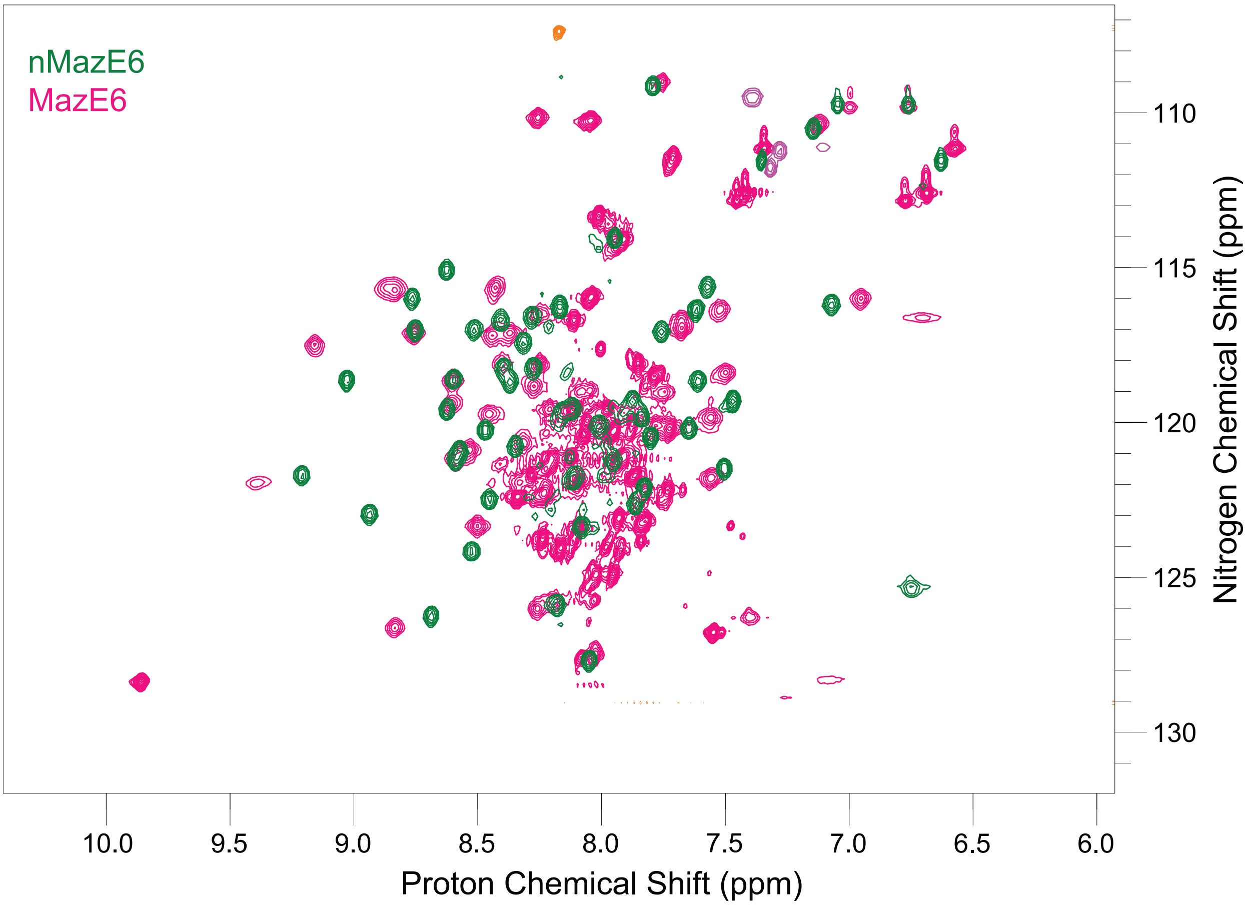

Figure S6

Figure S6: Comparision of nMazE6 with MazE6 An overlay of HSQC of MazE6 with HSQC of nMazE6. Peaks in nMazE6 overlaps well with that of peaks in well-dispersed region of MazE6. Shifts are seen for few residues due to presence of residual N-terminal His$_6$-tag and absence of C-terminal domain in nMazE6.

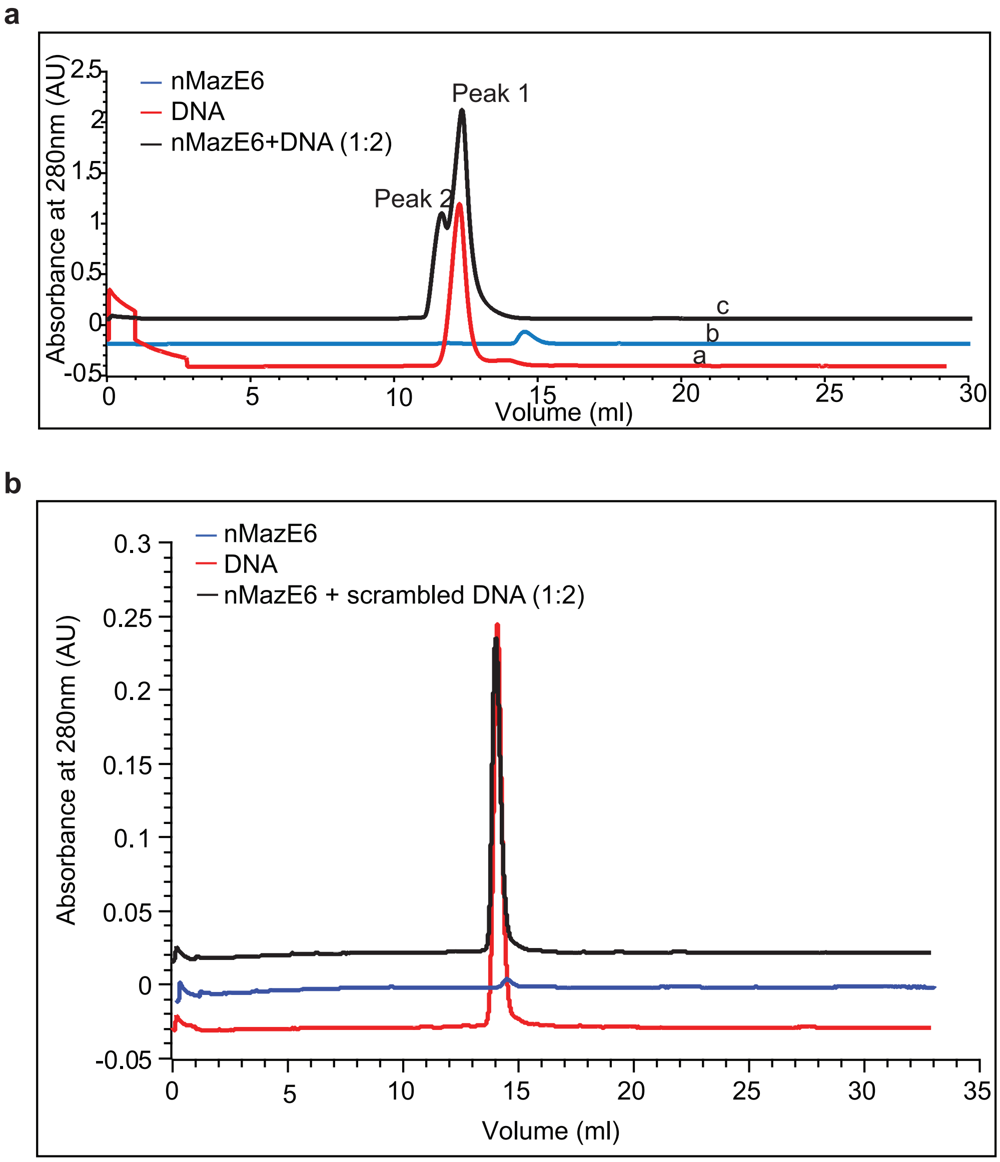

Figure S7

Figure S7: Interaction of nMazE6 with operator DNA: (a) Stacked plot of gel-filtration chromatograms of *mazEF6* operator DNA (red), nMazE6 (blue) and nMazE6+DNA (1:2) (black). Comparision of the chromatograms shows that Peak1 and Peak2 in chromatogram (c) corresponds to DNA and nMazE6-DNA complex respectively. **(b)** Stacked plot of gel-filtration chromatograms of scrambled DNA (red), nMazE6 (blue) and nMazE6+scrambled DNA (1:2) (black). Comparision of the chromatograms show that no complex has formed. Superdex-75 analytical column (24mL) was used to do the analysis. Absorbance axes have been offset for clarity.

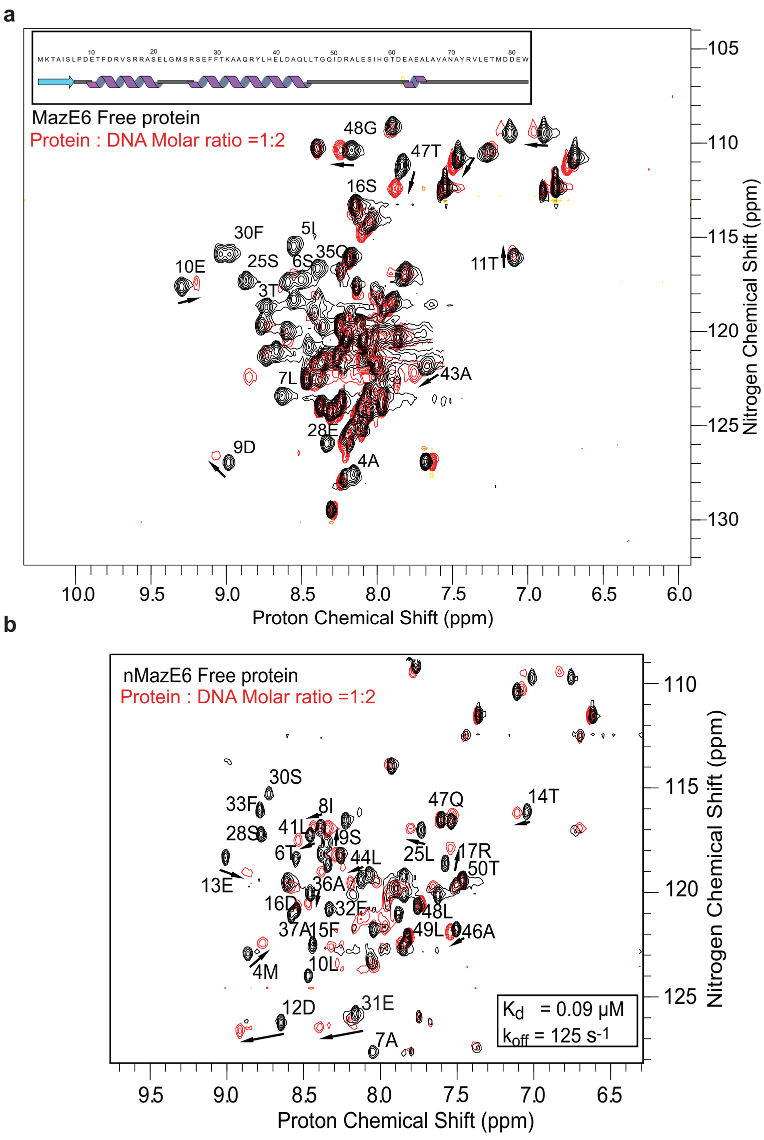

Figure S8

Figure S8: Chemical Shift Perturbation (CSP) upon cognate operator DNA binding: (a) Chemical shift perturbations observed in the ^{1}H- ^{15}N HSQC of MazE6 upon titration with *mazEF6* operator DNA at a 1:2 (MazE6:DNA) molar ratio. Spectra of free MazE6 (Black) and MazE6 in complex with *mazEF6* operator DNA at a ratio of 1:2 (Red) are shown. **(b)** CSP observed in the ^{1}H- ^{15}N HSQC of nMazE6 upon titration with operator DNA at a 1:2 (nMazE6:DNA) molar ratio. Spectra of free nMazE6 (Black) and nMazE6 in complex with operator DNA at a protein:DNA ratio of 1:2 (Red) are shown. Changes in chemical shifts of backbone amide resonance position between the first and last titration points are indicated by black arrows.

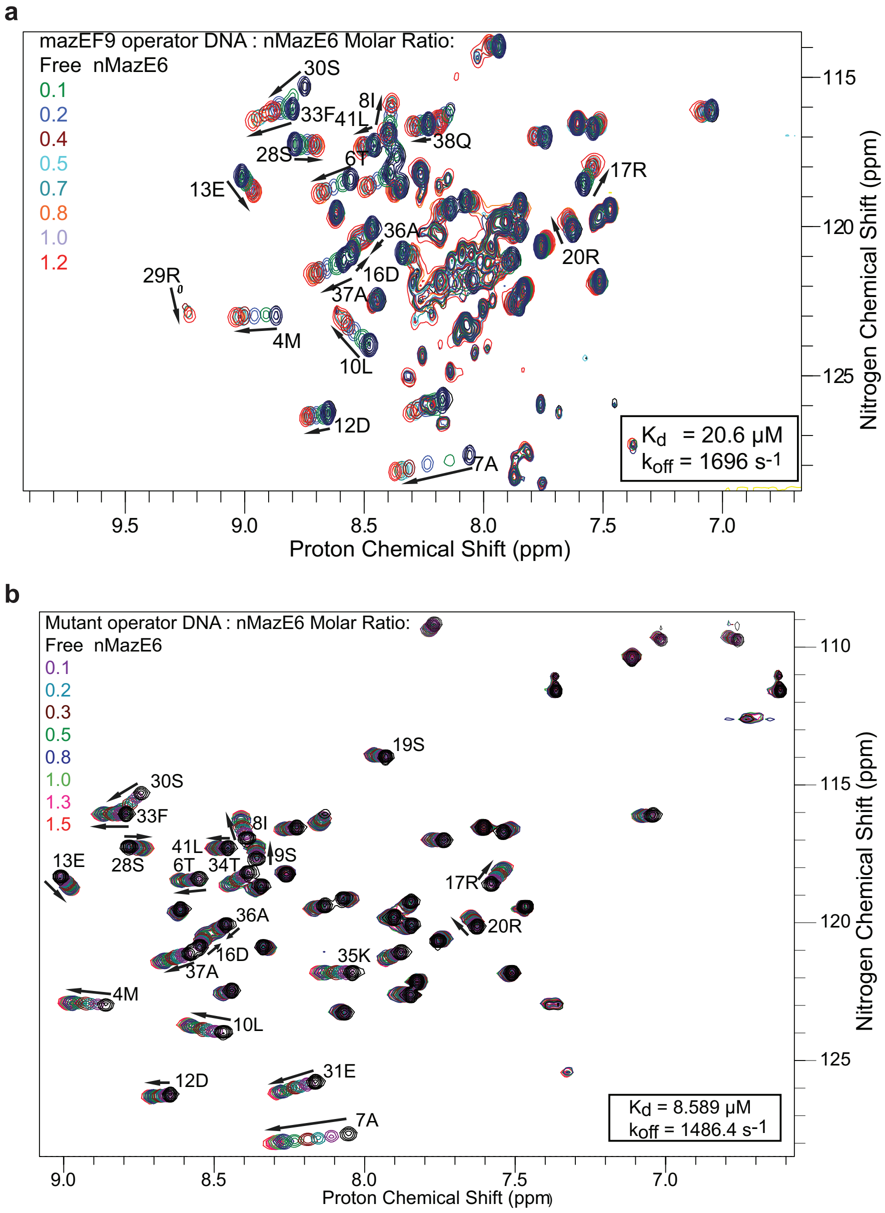

Figure S9

Figure S9: Chemical Shift Perturbation (CSP) upon non-cognate and mutated operator DNA binding: **(a)** Chemical shift perturbations observed in the ^1H-^{15}N HSQC of nMazE6 upon titration with *mazEF9* operator DNA. Changes in chemical shifts of backbone amide resonance position between the first and last titration points are indicated by black arrows. **(b)** Chemical shift perturbations observed in the ^1H-^{15}N HSQC of nMazE6 upon titration with mutated operator DNA. Changes in chemical shifts of backbone amide resonance position between the first and last titration points are indicated by black arrows. Mutated DNA sequence: 5′-CCGGTTA<u>GCA</u>TATCTGTA-3′

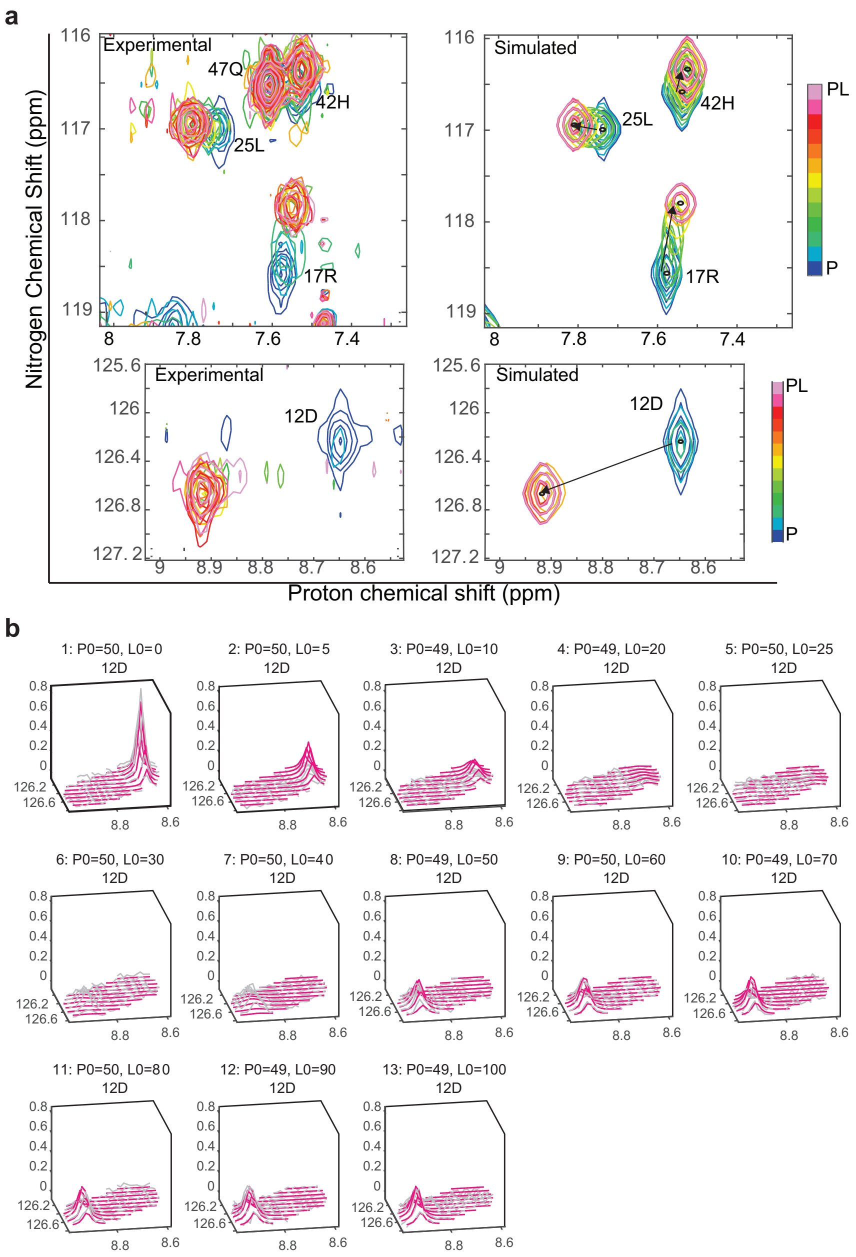

Figure S10

Figure S10: Two-dimensional lineshape analysis of nMazE6 upon titration with *mazEF6* operator DNA: (a) Experimental and fitted ^{1}H-^{15}N HSQC spectra of few residues. The chemical shifts of free and bound states determined by the fitting procedure are marked by the tail and head of the arrows shown in the fitted spectra. **(b)** Comparision of experimental (grey) and fitted (magenta) data for the residue 12D of nMazE6 at all the ligand (operator DNA) concentrations. Simulated lineshapes agree well with the experimentally observed lineshapes.
